# The Leucocyte in Disease.—II

**Published:** 1896-04-25

**Authors:** H. Wansbrough Jones


### THE LEUCOCYTE IN DISEASE.—II.

By H. Wansbrough Jones, M.B., C.M.Edin., B.Sc. Lond.

(*Continued from Vol. XIX. page* 325.)

Cohnheim, in 1867, first satisfactorily explained the important part played by leucocytes in the process of inflammation. He showed that, as the result of a local irritant, there is an invasion of the part by leucocytes, which leave the blood-vessels and rapidly infiltrate the tissues, disappearing again if the inflammation subside, or undergoing degeneration if suppuration occur. But it was not until it was known that bacteria or their products are among the commonest and most deadly irritants, and Metschnikoff's work on the pathology of inflammation was published, that the real processes at work began to be understood. Metschnikoff has shown that the lowliest of protozoa have to struggle against infectious organisms, and that if they succeed in overcoming them, it is by ingesting and digesting them; that, passing upwards, animals like the arthropods or mollusca can defend themselves by means of their leucocytes against infection by saprophytes, to which animals like the lepidoptera, hymenoptera, or diptera, which are poor in blood and leucocytes, rapidly succumb; that both in these animals and the vertebrata the actual process of phagocytosis can readily be observed. He has described minutely this process of phagocytosis in every kind of immunity, and concludes that it is not only *one* but *the* cause of immunity.

I do not propose to discuss this question here further than to say that those who oppose these views affirm that phagocytosis may occur without immunity, and that immunity may be obtained artificially without phagocytosis. I wish rather to draw attention to some other aspects of inflammatory leucocytosis. It was stated in the first of these papers that the coarsely granular eosinophile cell is not phagocytic. Does it, then, take any part in the reaction against a local irritant? The evidence on the subject is still somewhat conflicting. Sherrington found that in producing a local inflammation by a non-bacterial poison, there is first a diminution and then an increase in the number of leucocytes in the blood; that both diminution and increase affect chiefly the finely granular eosinophile cells, and that the coarsely granular eosinophile cells practically disappear from the circulation. On the other hand, Canon and others have found considerable increase of coarsely granular eosinophile cells in skin diseases; and while they are certainly found in large numbers at the seat of irritation in many conditions, this is not invariably the case. Thus they have been observed at the seat of local irritation by anthrax in the frog, in fresh gonorrheal pus, in tubercular joint disease after tuberculine injection. Kanthack says that they are generally to be found in blisters, vaccinia pustules, and the bullæ of pemphigus. I have constantly examined these fluids, but seldom found any but the finely granular cells, with granules stained deeply. Still this is a case where a little positive evidence is worth more than a great deal of negative evidence, so that the observation made by Kanthack and Hardy, that after the introduction under the skin of frogs of anthrax material the coarsely granular non-phagocytic cells appear some time before the finely granular cells, and that these latter straightway swallow up the bacilli, would seem to establish the importance of the action of these cells.

Kanthack thinks that there are "two stages in the conflict between wandering cells and bacteria—(*a*) The stage of an eosinophile attack; (*b*) the stage of a phagocytic ingestion"; and that in the earlier stages of local irritation the coarsely granular eosinophile cells are more powerfully attracted. It is supposed by many that the granules are of the nature of protective substances, which destroy the bacteria or neutralise their products, but Metschnikoff and Gulland consider them as merely nutritive material, and believe that the physiological activity of the cells will explain their action. The next point to be considered is, what is it that attracts the leucocytes to the seat of irritation and causes a diminution in the number in the blood? In 1884 Stahl discovered that solutions of dead leaves attract, while saline solutions repel organisms. Pfeffer more especially worked at this subject, and found that there is a general attraction to useful substances, *e.g.*, food, a repulsion from harmful substances; but he found that spirilla which were repelled by too concentrated solutions of glycerine or sugar would rush to the same if attractive substances were added, and perish in consequence. To these conditions of attraction and repulsion he gave the name of positive and negative chemiotaxis. It is, of course, possible that a positive chemiotaxis will explain the rush of leucocytes to an irritated spot, though it is difficult to see why one kind should be attracted before another. It is not, however, suggested by anyone—and, indeed, is known to be contrary to fact—that there is any proportion between the number of leucocytes which appear in an inflamed area and the number which disappear from the circulation; on the contrary, it is supposed that the leucocytes which disappear under such conditions collect in the liver, lung, spleen, and marrow, and are attracted again into the blood by a process of positive chemiotaxis. Goldsheider and Jacob have proved that there is such an increase in the lungs on producing such conditions, but this is the only positive evidence on the subject. It is certain, on the other hand, that leucocytosis may occur without chemiotactic substances, as the result of substances which Metschnikoff calls stimulines, so that it may be taken as proved that chemiotaxis, as it is usually interpreted, will not explain this alternate diminution and increase in the number of leucocytes. I think it more probable that there is an initial destruction—a leucolysis—and that the substances liberated by this breaking down act as stimulines, and produce a multiplication of the leucocytes already in the blood and blood glands.

And this brings me to the last point: (3) What becomes of the leucocytes engaged locally at the seat of irritation, and those which disappear from the circulation? Is there such a process as leucolysis? The answer to the first part of the question may be given in Metschnikoff's words: "Many cells perish, and are swallowed by other phagocytes. Some pass into the lymphatics, and so circulate again. Some become fixed cells." The evidence in favour of leucolysis is, to my mind, not inconsiderable, though doubt is still felt in many quarters as to its existence.

The word was first used by Löwit. I shall discuss this subject by first trying to determine what would become of leucocytes which broke down and dissolved in the blood; and, secondly, what evidence there is that such processes do take place in the conditions under consideration.

It was stated in the first of these papers that the albuminoid constituents of leucocytes are tissue fibrinogen, paraglobulin, and other cell proteins, and that this tissue fibrinogen is a nucleo-albumen, splitting up into a protein variety and a non-protein nuclein variety. Horbaczewski has shown that xanthin bases and uric acid can be obtained from nuclein by simple chemical changes. Now it has been demonstrated abundantly that wherever there is a considerable disintegration of leucocytes in the body, the urine shows traces of albumose and excess of uric acid. This has been found in cases of pus absorption, pyæmia, prolificating tumours of bone marrow, and in resolving pneumonias. Again, Wright has shown that if a solution of cell fibrinogen be injected into the blood of animals, albumosuria and increased excretion of uric acid invariably result. A similar increased excretion of uric acid has been constantly, and albumosuria occasionally, observed in fevers during the stage of diminution in the number of leucocytes. I have noticed both these symptoms in cases of gout, and have notes of the case of a lady whose leucocytes proportion was increased to the extent of 1 to 50 to 1 to 130, but varied considerably from week to week, in which increased excretion of uric acid was sometimes accompanied by albumosuria, sometimes by traces of albuminuria. These facts argue strongly in favour of leucolysis. The positive evidence is certainly not conclusive, but there is some. Wright has proved that the injection of peptone into dog's blood caused a diminution in the number of leucocytes from 17,725 to 1,560 per c. mm. These missing leucocytes must either have passed into solution, or disappeared into the tissues or internal vessels. Wright further made comparative estimations of the leucocytes in the mesenteric veins and carotid arteries, and made histological examinations of various organs, but in no case found evidence either of "stain or emigration of leucocytes either in the dog or rabbit after peptone injections." He believes, then, that the leucocytes do pass into solution, and thinks that this view is supported by the consideration that peptone placena deposits large quantities of nucleo-albumen on cooling. It may at any rate be safely argued, then, that leucolysis is probable, and it is certain that it offers the best explanation of the facts that have been under consideration.

I have tried in these short papers to show how much has been added in recent years to our knowledge of the leucocyte in disease. It would be easy in one's enthusiasm to believe that we have in some of these results the key to the explanation of the most perplexing problems of disease. Suggestions of one's own views would be useless. We can only wait for more facts.

Works Consulted.—Wright, Proc. Roy. Irish Acad., 3rd Series, Vol. ii., No. 2; Proc. Roy. Soc., Vol. lii. Sherrington, Proc. Roy. Soc., Vol. lv. Kanthack and Hardy, Proc. Roy. Soc., Vol. lii. Gulland, Development of Lymphatic Glands. Metschnikoff, Comparative Pathology of Inflammation. Horbaczewski, M. J. Chemie, &c., Vienna, 1891, Vol. xii.

